# Supporting the improvement of air quality management practices: The “FAIRMODE pilot” activity

**DOI:** 10.1016/j.jenvman.2019.04.118

**Published:** 2019-09-01

**Authors:** E. Pisoni, C. Guerreiro, S. Lopez-Aparicio, M. Guevara, L. Tarrason, S. Janssen, P. Thunis, F. Pfäfflin, A. Piersanti, G. Briganti, A. Cappelletti, I. D'Elia, M. Mircea, M.G. Villani, L. Vitali, L. Matavž, M. Rus, R. Žabkar, M. Kauhaniemi, A. Karppinen, A. Kousa, O. Väkevä, K. Eneroth, M. Stortini, K. Delaney, J. Struzewska, P. Durka, J.W. Kaminski, S. Krmpotic, S. Vidic, M. Belavic, D. Brzoja, V. Milic, V.D. Assimakopoulos, K.M. Fameli, T. Polimerova, E. Stoyneva, Y. Hristova, E. Sokolovski, C. Cuvelier

**Affiliations:** a European Commission, Joint Research Centre (JRC), Directorate for Energy, Transport and Climate, Air and Climate Unit, Via E. Fermi 2749, I-21027, Ispra, VA, Italy; b NILU Norwegian Institute for Air Research, Instituttveien 18, 2027 Kjeller, Norway; c Earth Sciences Department, Barcelona Supercomputing Center, Barcelona, 08034, Spain; d VITO, Flemish Institute for Technological Research, Boeretang 200, 2400 Mol, Belgium; e IVU Umwelt GmbH, 79110 Freiburg, Germany; f ENEA, National Agency for New Technologies, Energy and Sustainable Economic Development, Laboratory of Atmospheric Pollution, Bologna-Ispra-Pisa-Roma, Italy; g Slovenian Environment Agency, Ljubljana, Slovenia; h FMI, Finnish Meteorological Institute, Helsinki, Finland; i HSY, Helsinki Region Environmental Services, Helsinki, Finland; j Environment and Health Administration, City of Stockholm, Sweden; k ARPAE Emilia Romagna, Bologna, Italy; l Irish Environmental Protection Agency, Ireland; m Institute of Environmental Protection - National Research Institute, Poland; n Minister for Environment, Croatia; o Meteorological and Hydrological Service, Croatia; p Institute for Environmental Research and Sustainable Development, National Observatory of Athens, Lofos Koufou, 152 36 Penteli, Greece; q Warsaw University of Technology, Poland; r Institute of Geophysics, Polish Academy of Sciences, Poland; s “Climate, Energy and Air” Directorate, Sofia Municipality, USA; t Universität für Chemische Technologie und Metallurgie, Sofia, USA; u Ex European Commission, Joint Research Centre, Ispra, Italy

## Abstract

This paper presents the first outcomes of the “FAIRMODE pilot” activity, aiming at improving the way in which air quality models are used in the frame of the European “Air Quality Directive”. Member States may use modelling, combined with measurements, to “assess” current levels of air quality and estimate future air quality under different scenarios. In case of current and potential exceedances of the Directive limit values, it is also requested that they “plan” and implement emission reductions measures to avoid future exceedances. In both “assessment” and “planning”, air quality models can and should be used; but to do so, the used modelling chain has to be fit-for-purpose and properly checked and verified. FAIRMODE has developed in the recent years a suite of methodologies and tools to check if emission inventories, model performance, source apportionment techniques and planning activities are fit-for-purpose. Within the “FAIRMODE pilot”, these tools are used and tested by regional/local authorities, with the two-fold objective of improving management practices at regional/local scale, and providing valuable feedback to the FAIRMODE community. Results and lessons learnt from this activity are presented in this paper, as a showcase that can potentially benefit other authorities in charge of air quality assessment and planning.

## Highlights

• Air quality is still a challenge in various areas in Europe.
• FAIRMODE provides methodologies and tools for supporting air quality improvement.
• Specifically, a new FAIRMODE initiative for better management practices has been recently launched.
• The initiative provides a “live tutorial” to interested authorities.
• First results on improved emission inventories and concentrations are presented.

## 1 Introduction

As stated by the World Health Organization (WHO) and the European Environmental Agency (EEA), air pollution is the biggest environmental risk to health in the European Union (EU), causing more than 400.000 premature death in Europe every year. Thus, there is a need to improve air quality, and as the majority of people live in cities, it is crucial to focus on urban areas to tackle this challenge. From the legislative point of view, the 2004 and 2008 Air Quality Directives (2004/107/EC and 2008/50/EC) regulate air quality in Europe. They set standards for concentrations of pollutants in the air, while the 2008 Air Quality Directive specifically requires Member States to assess air quality and design and implement plans in case of exceedances of limit values. Moreover, since December 2016 the National Emissions Ceilings (NEC) Directive (2016/2284/EU) requests that Member States report regularly their emissions and make air pollution control programs in order to improve air quality. Thus, in the NEC context, also the importance of updated and detailed emission inventories is underlined, for understanding the relationship between source and receptor before deciding on measures to improve air quality.

In spite of having the legislation in place, in 2016 twenty-six out of twenty-eight European Union countries yet failed to comply with at least one of the limit values set by the EU directives, in particular for PM (EEA, 2018). Furthermore, as recently stated by the European Court of Auditors (ECA, 2018), part of the problem is linked to the fact that some “Air Quality Plans are not designed as effective tools”. Three main reasons that compromise the effectiveness of air quality plans were identified by the Court of Auditors:

1. Plans are not targeted, and cannot be implemented efficiently in the areas where the highest concentrations were measured;
2. Plans can't deliver significant results because they went beyond the jurisdiction of the local authorities responsible for their implementation or because they were designed for the long term;
3. Plans were not supported by cost estimates analysis or were not funded.

Models (Pisoni et al., 2019) are an essential tool in the context of preparing “targeted” plans (Carnevale et al., 2014). A pre-requisite for their application is that the used modelling chain (encompassing emission inventories, air quality models, etc …) is robust and well validated.

In this paper, we present the on-going activities in the FAIRMODE community to try to improve this chain, as well as the way air quality assessment and planning are performed. FAIRMODE is the European Forum for Air Quality Modelling, created for exchanging experience and results (from the air quality modelling point of view) in the context of the Air Quality Directives (AQD) and for promoting the use of modelling for air quality assessment and management in a harmonized manner between Member States. In particular, the Air Quality Directive encourages the use of model:

- As a tool to supplement monitoring data for assessment purposes when reporting exceedances of air quality limit values;
- To prepare action plans to improve air quality, both in the short and long term.

Specifically to address the issue of the Air Quality Directive compliance, and to support the regional/local authorities in the improvement of their air quality assessment and management practices, the FAIRMODE ‘pilot initiative’ was launched. A group of interested regional and local authorities was created with the aim of establishing, from the users point of view, to what extent the FAIRMODE methodologies and tools can help improving modelling applications.

So, the ambition of the ‘pilot initiative’ is:

1. To provide a live tutorial/training to interested regional/local authorities on harmonized methodologies and tools to be applied to improve their emission inventories, air quality modelling assessment and subsequently their air quality plans preparation;
2. To improve the quality and usability of the FAIRMODE tools, based on the feedback from the participants' pilots, to better address the regional/local authorities' needs.

At the end of the pilot activity, FAIRMODE ideally will have a coherent protocol to be used by regional and local authorities, as well as researchers/air quality specialists, to benchmark and improve their modelling applications. The innovative aspect of the FAIRMODE pilot activity is the creation of a common platform to analyse data and present informed solutions specific to the local characteristics of each participant using some common methodologies and statistical tools developed specifically for this purpose (for more information please refer to Thunis et al., 2012a, b).

The paper presents case studies linked to emission inventories and assessment of air quality model applications, and the first lessons learnt during the ‘pilot initiative’. The manuscript is structured as follows. After a brief presentation of FAIRMODE, the tools and the objectives of the ‘pilot initiative’, we will detail the lessons learnt during the first phase of the work. Finally, as an illustrative example, we will show how a city or region can apply the proposed FAIRMODE set of tools to benchmark and improve its modelling application.

## 2 The FAIRMODE community and tools

### 2.1 The FAIRMODE community

FAIRMODE is the Forum for Air Quality Modelling created to exchange experience and results in the context of the AQD. The activities of this network have been linked to assessment, guidance and benchmarking of modelling applications, including the improvement of methods for air quality assessment modelling and forecasting, developing emission inventories, source apportionment and air quality management and planning.

### 2.2 FAIRMODE main objectives are

- To provide a permanent European Forum for air quality modellers, particularly addressing harmonization and quality of model applications;
- To study and set-up a system (protocols and tools) for the quality assurance of air quality models operating at different spatial scales from national to urban and local;
- To support a standardized evaluation of the fitness-for-purpose of air quality models and input data, for assessing current and future air quality within the framework of implementing the EU's Air Quality Directives;
- To support air quality management (at the national, regional and local level) in developing and implementing plans and measures to improve air quality with efficient tools;
- To promote capacity building activities aiming at ensuring an optimum use of the proposed common methodologies and guidance and to promote good practice among Member States;
- To make recommendations on future priorities, research activities and other relevant initiatives to secure air quality improvements.

On top of this main objectives, FAIRMODE developed an implementation strategy. The FAIRMODE ‘implementation strategy’ is based on the so-called FAIRMODE ‘wheel of knowledge”. The strategy is to proceed through a three-step process based on 1) Benchmarking, 2) Guidance and 3) Capacity building and Communication. Benchmarking is intended here as the compilation of different approaches and the subsequent development and testing of a standardized evaluation/inter-comparison methodology for collecting and reporting model inputs/outputs in a way that enables relevant comparisons. The aim is to identify good practices and propose ways to diagnose problems in performance. Once a common evaluation/inter-comparison methodology is agreed upon, guidance documents can be drafted setting the path to capacity building with the overall objective of promoting good modelling practices in member states. Communicating these good practices identified by expert groups to the broader community that includes also national and local authorities in charge of the application of models for regulatory purposes, is also a key issue.

Since its start FAIRMODE has developed and introduced various methodologies and tools to benchmark and support air quality modelling. The key tools used in the present paper are the DELTA-emis-tool and DELTA-conc-tool (already presented in previous publications, and shortly described in the Supplementary Material) and the “Composite Mapping Tool”, presented in the next section.

### 2.3 The composite mapping tool

The ‘European Composite Mapping’ tool (http://fairmode.jrc.ec.europa.eu/ecmaps/) complements the analyses provided by the DELTA tools with visual inspection for qualitative and quantitative (spatial) comparison of ambient air concentration and emission maps. In particular, the ‘Composite Map’ offers a mosaic of the best available national, regional or local estimates. The main objectives of the tool are capacity building and triggering discussions on topics such as:

• border effects between neighbouring regions/countries and the reasons behind observed differences (e.g. methods, input data, resolution);
• quality and consistency of emission inventories and concentration maps;
• use of data assimilation or data fusion techniques to produce air quality maps;
• representativeness of the spatial proxies used to spatially distribute emissions;
• choice of an adequate domain and spatial resolution for a particular application.

For the concentration maps, NO2 and PM10 data were collected as annual mean concentration values with a focus on the years 2012 and 2015. For the emissions, data was collected as annual total values for the main precursors (NOx, NMVOC, CO, SOx, NH3, PM10 and PM2.5) and per SNAP sectors (Selected Nomenclature for sources of Air Pollution), focusing on 2015. Both emission and concentration maps are provided as gridded data in ASCII or GeoTiff format. This data format restriction is important to avoid the exchange of exotic file formats and subsequent misinterpretations of the data in the visualization process. During the exercise it turned out that the processing and interpretation of the data was the most labour intensive step in the whole exercise. To reduce the risk of misinterpretation (e.g. on projection systems), a “Quality Check Tool” was developed. All data sets had to pass this quality check before they could be uploaded into the Composite Mapping data base. The main quality control checks relate to file format, projection systems, geographical referencing, missing value labels and internal data consistencies (e.g. minimum and maximum values within reasonable ranges). When the quality control was successfully passed, the data could be uploaded into FAIRMODE's Composite Mapping database. During the upload process, data providers also supplied the relevant metadata information, e.g., contact information, model name and type, model output frequency and type of data assimilation.

Once stored in the database, the maps can be visualized and analysed by a web based platform made available via the FAIRMODE website (http://fairmode.jrc.ec.europa.eu/ecmaps/). The platform allows zooming into specific domains, enabling or disabling individual maps, modifying the colour legend and drawing a concentration or emission profile along a user defined linear transect. An example of such a concentration profile is given in Figure A (Supplementary Materials). The concentration profile is useful to explore for example concentration differences and inconsistencies at inter-regional or inter-national borders. For the analysis of emission maps an additional functionality was developed, which allows the spatial comparison of different emission inventories and averaged over a user defined polygon. In this way the consistency between two of more inventories for a specific region can be analysed.

Over 60 maps are available in the Composite Concentration map including national, regional and urban air quality maps covering a large part of Europe. In addition, the air quality maps provided by the European Environmental Agency's (EEA) Topic Centre on Air pollution and Climate Mitigation (ETC/ACM) are included as a European wide benchmark as well as monitoring data for the year 2012 and 2015 from EEA's Air Quality e-Reporting Database. For the latter one, the platform offers the opportunity to select various types of stations so that a proper and representative comparison can be made with the modelled results.

The emission platform also contains national, regional and local emission inventories for the main precursors, classified according to the SNAP nomenclature. As EU wide benchmarks the emission inventories provided by EMEP (officially reported emissions to the Convention for Long-Range Transboundary Air Pollution (CLRTAP; http://www.unece.org/env/lrtap/welcome.html), CAMS/MACC (used in the Copernicus Services, https://atmosphere.copernicus.eu/) and the JRC (a more ad-hoc emission inventory for JRC-related activities) are included.

## 3 The pilot initiative

This section presents more in detail the FAIRMODE pilot activity. The ‘pilot’ work-plan covers two distinct phases: “assessment” and “planning” (Thunis et al., 2015). This manuscript focuses on the assessment phase (including emission and concentrations), as the planning phase is still in preparation. In a later stage, also the planning phase should go through the FAIRMODE tools applications, as i.e. using the SHERPA tool (Clappier et al., 2015; Thunis et al., 2016a, Thunis et al., 2016b; Pisoni et al., 2017), and applying source apportionment.

### 3.1 Main objectives

The main aim of the pilot initiative is to test the methodological approaches developed in FAIRMODE, promoting their use to support and improve air quality management practices at the regional and local scale. In the framework of this activity, pilot's participants will apply the FAIRMODE tools to their specific domain, ideally improving their current approach (through the use of common tools) and at the same time providing feedback to FAIRMODE on the usefulness of the proposed tools.

Specific objectives of the pilot initiative are:

• Supporting the participants in the improvement of their current practices, through the use of FAIRMODE tools
• strengthening the links between FAIRMODE and the local and regional authorities;
• improving the FAIRMODE support based on the participants' feedback.

To ensure efficient interactions between the pilot regions/cities and FAIRMODE, it is required that the participants have data and modelling tools available for assessment and planning purposes. In particular, an air quality model, gridded and sectoral detailed emission data as well as monitoring data of the pollutants requested by the AQD. For the sectoral analysis of emissions, the pilot initiative focusses on the road traffic and residential combustion. Yearly and hourly monitoring data of PM10, PM2.5 and NO2 values are considered for concentration benchmarking.

The “assessment” phase consists in checking the quality of the air quality modelling chain by:

• checking the quality of emission inventories (or comparing emission inventories constructed with different methodologies with officially reported values to EMEP) via the methodologies developed in FAIRMODE by using the DELTA-emis-tool (Guevara et al., 2016);
• Participating in the “emissions composite mapping” exercise to check consistency with other maps for the same area and downscaled EU wide emission maps;
• Ensuring that the model applications fulfil the Modelling Quality Objective by using the DELTA-conc-tool (Thunis et al., 2012a, b);
• Participating in the “concentrations composite mapping” exercise, to check consistency with neighbouring maps or other maps for the same area.

### 3.2 Participants to the initiative

Table 1 shows the list with the pilot participants. As shown, the participants are from cities, regions/states, national authorities and research institutes; this guarantees the possibility to test, through different decision levels, how the FAIRMODE tools contribute to better air quality management strategies, and to get feedback from different points of view. Also, in Table 1 key pollutants and key sources (not only the one considered on this paper) are shown in the correspondent columns, to underline the scope of the work that the participants are tackling.

**Table 1:** Participants to the pilot initiative.

GOVERNMENT LEVEL	NAME	KEY POLLUTANTS	KEY SOURCES
CITY	Stockholm (SE)	NO2, PM10	Traffic and residential combustion
	Milan (IT)	NO2, PM10, PM2.5, O3	Traffic and residential combustion
	Dublin (IE)	NO2, PM10, PM2.5	Traffic and residential combustion
	Athens (EL)	NO2, PM10, PM2.5, O3	Traffic, residential combustion, navigation
	Sofia (BG)	PM10, PM2.5, B(a)P	Traffic (PM10, PM2.5), residential combustion (PM10, PM2.5, BaP)
	Helsinki (FI)	NO2, PM10, PM2.5, B(a)P	Traffic (NO2, PM10), residential combustion (PM2.5, BaP)
REGIONS/STATES	Emilia Romagna (IT)	NO2, PM10, PM2.5, O3	All sectors
	Malopolska (PL)	O3, NO2, SO2, CO, PM10, PM2.5	Residential combustion, traffic, industry
	Hessen state (DE)	NO2, PM10	Traffic, residential combustion, industry, biogenic
COUNTRY	Slovenia (SI)	PM10, O3	Residential combustion, traffic (PM10), regional (O3)
	Croatia (HR)	NO2, PM2.5, PM10	All sectors
	Italy (IT)	NO2, PM10, PM2.5, O3	Traffic, residential combustion, agriculture

Every participant to the initiative uses different models and spatial resolutions (see Table A, Supplementary Material, for details) and has different priorities and challenges (again, see Supplementary Material for details on priorities and challenges).

The next sections describe the lessons learnt from this activity.

## 4 Key lessons learnt from the first phase of the pilot initiative

We present here the main results from the assessment phase, structured around key lessons learnt based on the use of the aforementioned tools.

### 4.1 Lesson learnt 1: the need to identify inconsistencies between “downscaled” and “local” emission inventories

**Fig. 1:**
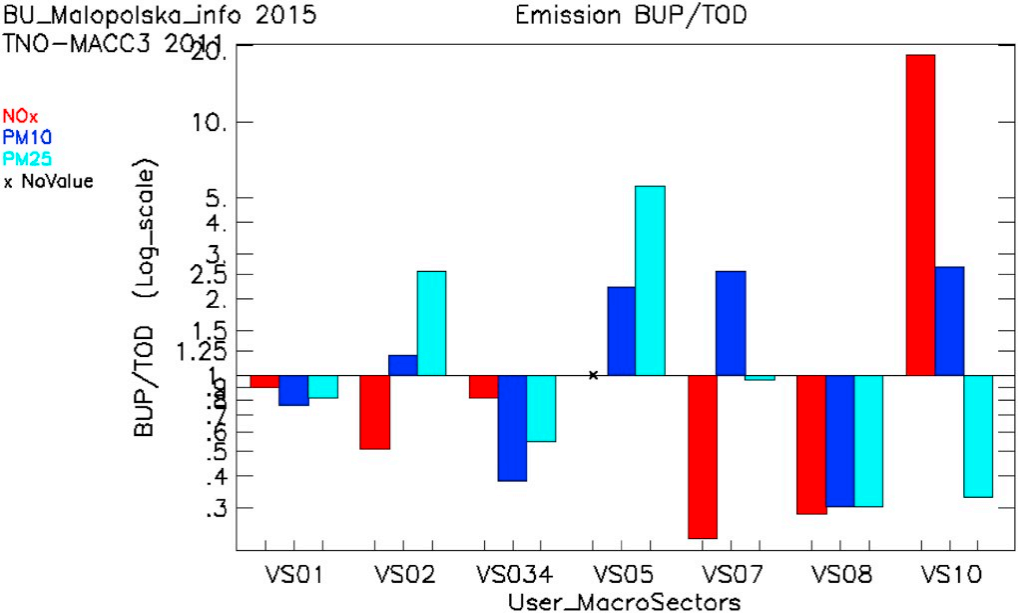
Application of the emission benchmarking tool, to quickly spot inconsistencies between TOD and BUP emission inventories. See Table B, supplementary Material for the definition of SNAP Macrosectors (defined as User_MacroSectors in Figure).

A first benefit of the application of the FAIRMODE tools, is to allow to quickly identify inconsistencies between European wide downscaled emission inventories (TOD, top-down, derived by total emissions per country gridded using proxies) and local/regional ones (BUP, bottom-up, derived starting from detailed information available at the local scale and aggregating it). An example of this issue is shown for the ‘Malopolska region’ domain. In Fig. 1 the BUP for 2015 is compared with TNO-MACC-III (Kuenen et al., 2014; a reference EU wide emission inventory) for 2011, on the same area. More in detail, Fig. 1 shows the total per SNAP macrosector^1^ (on the x-axis) and pollutants (colour) computed over the Malopolska regional domain, as a ratio between BUP and TOD.

Fig. 1 shows that there are indeed significant differences in BUP emissions vs. the TOD emission inventory. Sectors SNAP6 and SNAP9 are not included since their contribution to total NO_x_ and PM10/PM2.5 emissions is almost negligible. The differences between the inventories are not systematic within the same SNAP sector, as each pollutant can be driven by different processes (i.e. NOx traffic emissions are related to exhaust sources while PM10 is also linked to non-exhaust processes such as brake wear or resuspension). In this case for NOx in SNAP2 and SNAP7, the BUP/TOD ration is lower than 1. For PM10, BUP/TOD ratio is higher than 1, with relatively small difference in SNAP2 and the ratio factor reaching 3 in SNAP7. Even if the reasons for these differences cannot be explained by such type of Figure, indeed this type of ‘aggregated’ analysis can trigger further investigations to understand why these inconsistencies are found.

A further step in the analysis, to better understand the reasons for the aforementioned differences, can be done through the ‘diamond plot’ (Guevara et al., 2016), presented in Figure B (Supplementary Information). This type of analysis aims to provide an interpretation of the potential reasons behind the observed differences between TOD and BUP. The analysis in this case suggests that the differences may be related both to emission factors (x-axis) and to the estimated activity level (y-axis). For all data points within the red diamond, the emission factors, the activity rates and the emission totals differ at most by a factor of 2. The points outside the red diamond have higher differences. For the Malopolska domain, it should be underlined that a new BUP emission inventory has been prepared for the entire Poland and will be used for air quality modelling to support the air quality assessment for 2018. Emission analysis with FAIRMODE tools will be repeated with the new dataset.

### 4.2 Lesson learnt 2: the need for permanent improvement of emission inventories

**Fig. 2:**
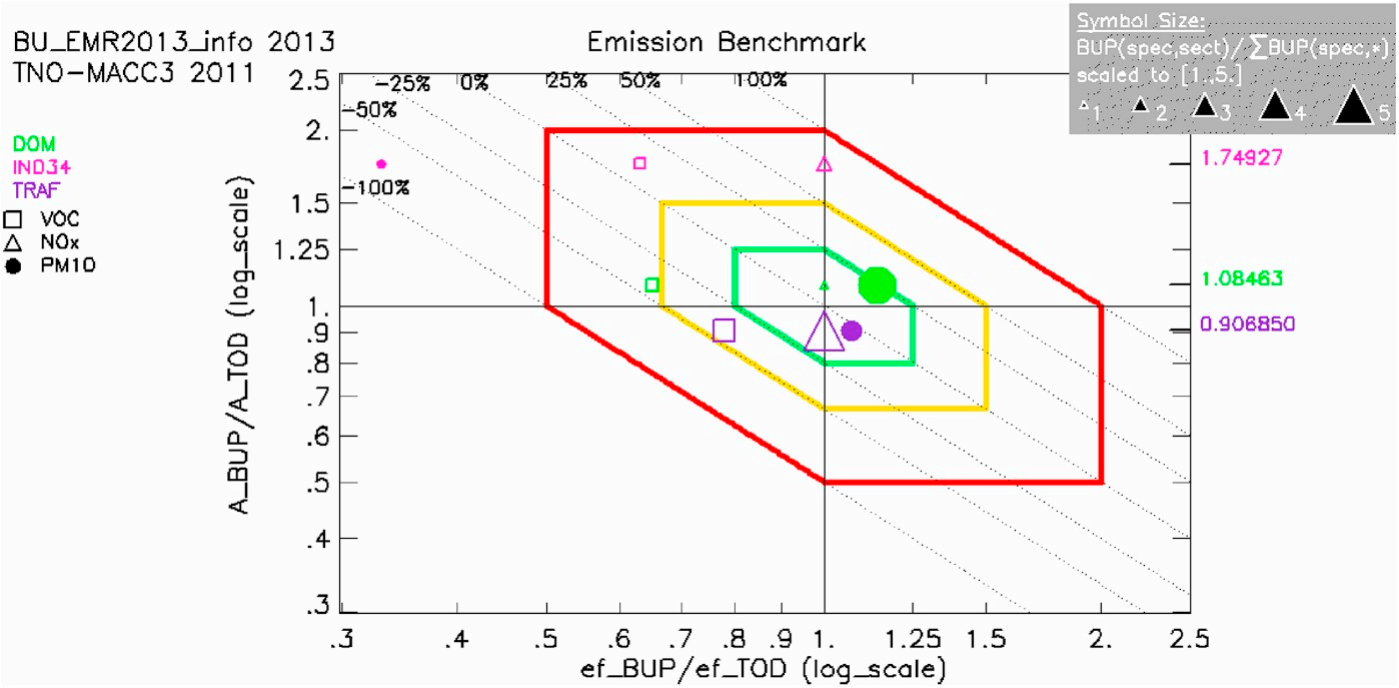
‘Diamond plot’ used to find an ‘emission factors issue’ for industrial sector.

Emission factors are a key information needed to building emission inventories (Fameli and Assimakopoulos, 2015). An in depth analysis on this issue was done by the Emilia Romagna region. In particular, for the Emilia Romagna case, a good agreement was reached between the local and downscaled inventories for domestic combustion and road traffic (Fig. 2). On the contrary, for the SNAP macro-sectors 3 and 4 (related to industrial emissions) the DELTA-emis-tool (Fig. 2) highlighted significant differences (i.e. PM10, for industry); but Fig. 2 cannot explain where this difference originates. To explain this difference, emission-associated metadata on the methods used to compile the two emission inventories were retrieved.

In particular, in regional (BUP) compilation of the emissions in SNAP macro-sectors 3 and 4, the Emilia Romagna region followed a detailed approach, measuring at the ‘stack’ (so with a high level of accuracy) emissions of the different pollutants. On the contrary the TOD (EU-wide emission inventory) is based on the national official emissions reported to the EMEP Centre on Emission Inventories and Projections (CEIP) (http://www.ceip.at/), which are mostly based on energy balances and fuel-depending emission factors. Most of the total national emissions are spatially distributed linking them to the emissions reported by E-PRTR, while the remaining are considered industrial diffusive emissions and subsequently distributed based on spatial proxies (i.e. industrial land uses). This means that there is a low ‘methodological alignment’ between the two inventories compared (that explains the differences) and in principle the ‘local’ measured emissions could be used to improve the TOD results. So this type of information could help to improve official reporting of emissions by CLRTAP; and also could foster the use of more accurate ‘local information’ to feed and improve the TOD emission inventory, i.e. providing updated emission factors for different geographical areas and sectors, or through publishing open-access data as done by US EPA with the continuous emission monitoring systems (CEMS), (https://www.epa.gov/air-emissions-modeling/emissions-modeling-platforms).

### 4.3 Lesson learnt 3: usefulness of higher spatial resolution

On top of the analysis on total emissions, FAIRMODE provides tools for the spatial analysis of emissions and concentrations. This is important, as local gridded information could be used to better detail/correct EU wide data. In particular, the composite mapping platform can be used to show spatial differences, as it offers the possibility to overlap and analyse different sources of data, and to check (among other things) the variability of the fields due to spatial resolution. Also the Concentration Composite Mapping platform allows to benchmark model results with observation from EEA's Air Quality e-Reporting Database. Note that such an ‘absolute’ benchmark is not possible for emissions.

**Fig. 3:**
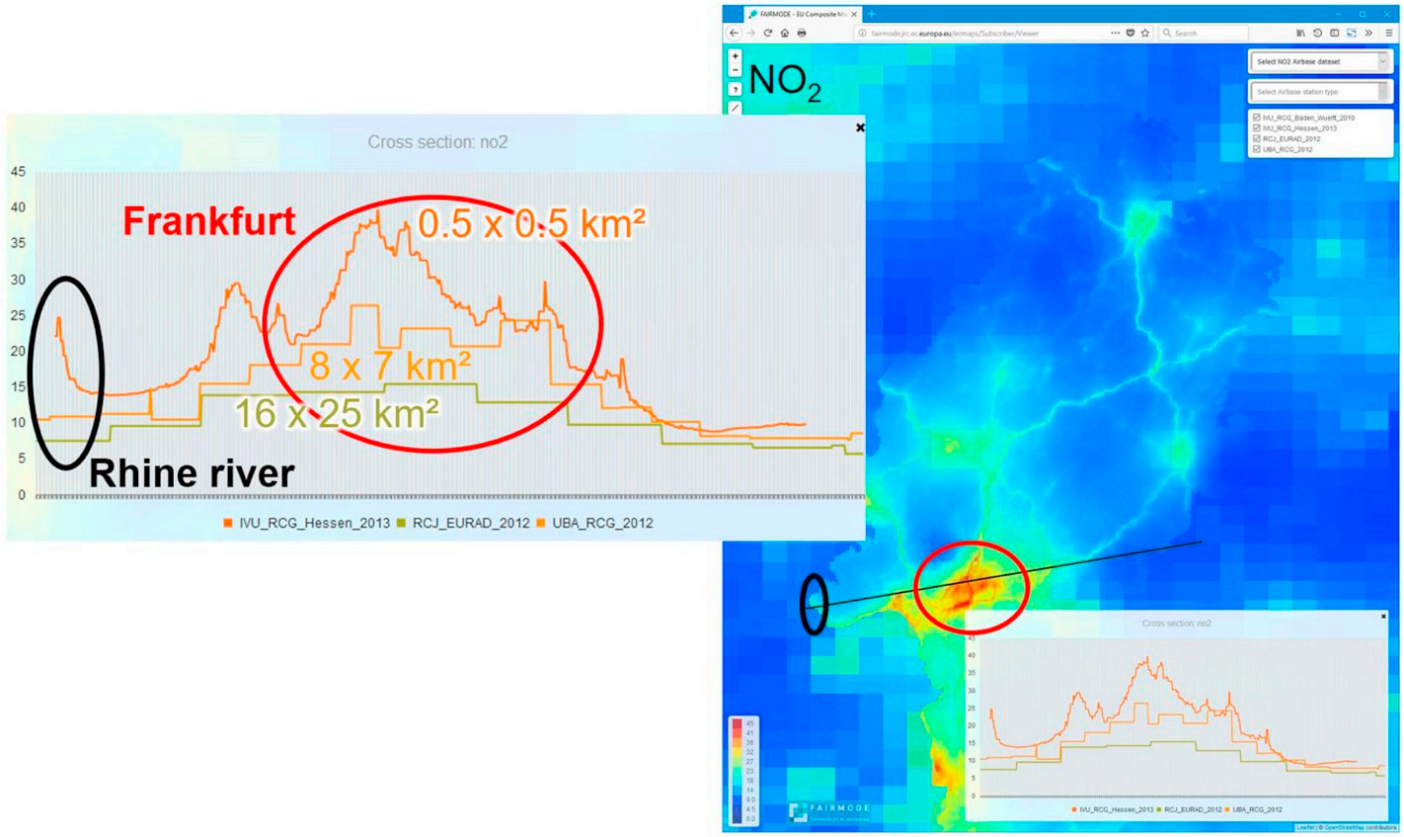
Checking resolution issues in concentrations maps, through the ‘composite mapping’ platform.

Fig. 3 demonstrates the effect of increased spatial resolution, for the Hessen state case (Pfäfflin, 2018) by means of three concentration maps with different resolutions (ca. 16 × 25 km^2^, ca. 8 × 7 km^2^ and ca. 0.5 × 0.5 km^2^). Given the transect line created in the composite mapping tool (black line, right side of the Figure), it is possible to appreciate the concentration variability (left side), showing increased values of NO2 moving from a background situation with a coarse resolution (green profile, lower concentrations e. g. in agglomerations) to a high resolution (dark orange profile, higher concentrations e. g. in agglomerations).

This in-depth view of concentration fields at different spatial resolutions can be used to better appreciate if low resolution model results are sufficient to properly describe the observed pollution levels, and when a higher resolution model is needed.

Apart from the resolution effect, the analysis can also reveal differences between model setups for the same region, which can be attributed to underlying emission inventories, boundary conditions or data assimilation strategies.

### 4.4 Lesson learnt 4: the importance of the model quality objective for concentrations benchmarking

A fourth lesson learnt is linked to the benchmarking of air quality model applications. In this context, the usefulness of the Modelling Quality Indicator and Modelling Quality Objective (Thunis et al., 2012a, b) has been confirmed in the application performed by Helsinki. The aim of the Helsinki metropolitan area pilot exercise was to study if the urban scale air quality modelling fulfils the quality requirements set for modelling of NO2 concentrations. The dispersion and chemistry model CAR-FMI (Contaminants in the Air from a Road - Finnish Meteorological Institute; Härkönen et al., 1995) was applied. CAR-FMI considers traffic-originated pollution from an open road network.

The Modelling Quality Indicator (MQI) can show if the model fulfils the defined model quality requirements. This indicator was computed for a period of 11 years, from 2004 to 2014. Concentrations of NO2 were studied altogether in 41 monitoring stations operated by Helsinki Region Environmental Services Authority (HSY). The number of stations available for each year varied from 5 to 10.

**Fig. 4:**
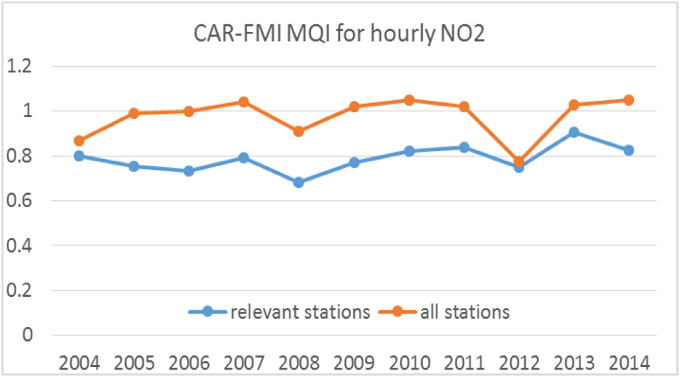
The MQI values for hourly NO2 during 2004–2014 with all stations (orange line) and with those relevant for CAR-FMI model, i.e. street canyons excluded (blue line).

The MQI value was computed for all study years with all stations and for all study years but only using those stations which are relevant for the open line-source model (i.e. street canyon stations excluded). The resulting MQI values for all study years are shown in Fig. 4. According to the MQI values, the CAR-FMI model fulfils the quality requirements (MQI < 1) in all years with all stations (in case stations, which are relevant for the model, are considered). But, as expected, the quality requirements were not fulfilled, (MQI > 1) in most of the cases when street canyon stations were included in the calculations.

More detailed information of the model performance at a single station can be examined from the Target Plots of the individual years. As an example, target plots for 2014 with all stations and with relevant stations (excluding street canyons) are shown in Figure C (Supplementary Materials). It can be seen that the model performance was worst for stations Mannerheimintie and Hämeentie, which both are street canyon stations. It is also possible to see that the performance of the model is relatively poor for Länsisatama station, located at the harbour (as ship and harbour emissions were not included in modelling).

Overall, the analysis in Figure C (Supplementary Materials) shows that all the points are located in the bottom left quadrant of the Target Plot. This means that the CAR-FMI model underestimates NO2 concentrations in 2014 in all stations (bottom quadrant, i.e. negative bias) and that the error associated with poor correlation dominates the one associated with the standard deviation (left quadrant). The same overall result was obtained for all study years.

The Target Plot combines different statistical features in a single diagram and lists the MQI of the modelling. The usage of different statistical indicators along with the possibility of identifying the dominant ones can be useful in order to point out the main sources of errors. So, the Target Plot gives useful information on the modelling performance and it helps to understand how the modelling performs for stations representing different environments and emission sources. However, the usability of the DELTA-conc-tool for several study years was not ideal. Allowing data of several years would enable more detailed study of the concentration levels and model performance. For example, the yearly concentration trends could be presented, more study locations could be included, and the model performance during different meteorological conditions could be studied. In addition, the conditions of the single year would not dominate on the general model performance result. Thus, the benefits of allowing the study of multi-year data, should be considered on the further development of the tool. Otherwise, this application confirmed that the Modelling Quality Indicator and Modelling Quality Objective provide a harmonized and useful benchmark for the evaluation of model results in the context of policy applications.

## 5 Following the whole loop: the Stockholm case

Looking at the work from another perspective, and after having checked the various lesson learnt, in this section we show how a given city (in this example, Stockholm) followed the whole pilot “assessment phase” for benchmarking and improving their emission and concentration estimation. Providing the application and analysis of all the tools for a specific city can be helpful to provide a sort of ‘template’ for other cities willing to test the FAIRMODE tools. The following subsections focus on emissions and concentrations analysis.

### 5.1 Emissions

Local emission data used for Stockholm in this study refers to the year 2015. The emission data is administered by the Air Quality Management Association of Eastern Sweden, and operated by SLB-analysis at the Environment and Health Administration, City of Stockholm. This local emission data is hereafter referred to as SLB emission inventory. In this study, we have focused on two source sectors, residential combustion and road transport.

The analysis in Figure D (Supplementary Materials) shows bar plots produced with the FAIRMODE DELTA-emis-tool for emissions (Δ-Emis). TNO-MACC-III and EMEP are used as TOD inventories, for comparison with the BUP. Note that the TNO-MACC-III emission data refers to the year 2011, whereas SLB local emissions and EMEP refers to the year 2015. For SLB and TNO-MACC-III the emission for road transport is provided, as well as emissions divided into exhaust emissions from petrol and diesel vehicles, and non-exhaust emissions. For residential combustion the local emission inventory is higher than both EMEP and TNO-MACC-III for PM10 emissions, whereas the opposite is true for NOx emissions. It is hard to draw any conclusions regarding the underlying causes of the observed differences between the different emission data sets of residential combustion. In any case, small scale domestic combustion is the source sector with the largest uncertainties in the local emission data, with major uncertainties in activity emission factors and spatial proxies used to grid data (Lopez-Aparicio et al., 2018). Depending on the quality of the emission data of EMEP and TNO-MACC-III the Delta tool comparison could help improve the local emission data, even if this will need a more in-depth knowledge of how the TOD are built (i.e., analysing the EMEP Informative Inventory Reports (http://www.ceip.at/ms/ceip_home1/ceip_home/status_reporting/2018_submissions/). NOx emissions from road traffic in Stockholm's local emission data are higher compared to EMEP and TNO-MACC-III; this could be due to a higher share of diesel passenger cars in Stockholm compared to the national vehicle fleet. In Sweden, there has been an increasing trend in the number of diesel cars at the expense of petrol vehicles during the last ten years, which could explain that the differences are even larger between SLB and TNO-MACC-III (year 2011) compared to the difference between SLB and EMEP (year 2015). In 2011, the share of diesel passenger cars in the Swedish national vehicle fleet was 17% compared to 26% in Stockholm County. For the year 2015, the national share of diesel passenger cars was 30% compared to 41% in Stockholm County.

In Sweden the emission of PM10 from road traffic is dominated by non-exhaust PM emissions. This is due to the use of studded tires/gravel/sand/salt during winter, which gives rise to large emissions of road wear PM. The emissions consist of both direct emissions and resuspension of road dust. Depending on e.g. meteorological conditions and road pavement the emission factor for resuspension of road dust can be even larger than the emission factor for direct emissions of road wear (Denby et al., 2013a, 2013b). In SLB local emission inventory, PM10 emissions from road traffic include PM-exhaust as well as both non-exhaust PM and resuspension from road wear; whereas in the Swedish inventory in 2011 only direct emissions of non-exhaust PM was considered. This explains the differences seen between EMEP and TNO-MACC in the FAIRMODE DELTA tool, i.e. SLB estimates lower PM10 emissions from road traffic compared to EMEP but higher compared to TNO-MACC-III. Looking at the meta information of the emission inventories (that is to say, how the emission inventories have been built), one possible explanation of why SLB has lower PM10 emissions than EMEP is that Stockholm has a lower proportion of vehicles with studded tires compared to the national average.

**Fig. 5:**
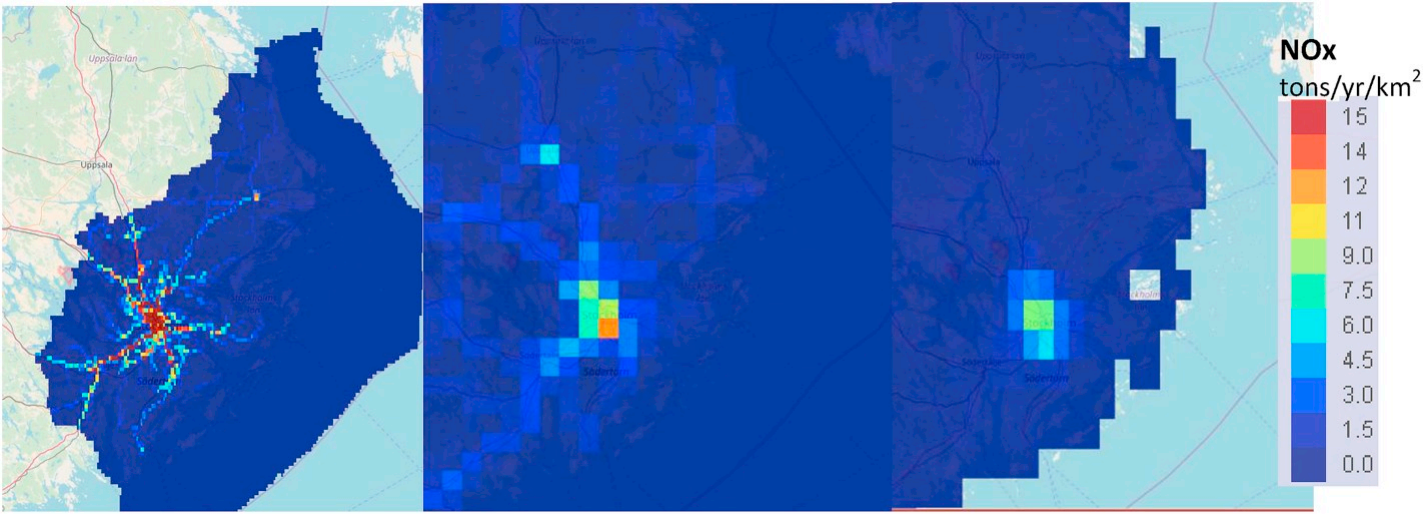
NOx road traffic emission maps for Stockholm County for (left) SLB, (centre) TNO-MACC-III and (right) EMEP. For TNO-MACC-III the emissions refer to the year 2011, for the other two it refers the year 2015.

Fig. 5 shows NOx road traffic emission maps for Stockholm County for three different data sets, SLB, TNO-MACC-III and EMEP. From the figure it is clear that the European downscaled data set cannot correctly describe geographical variability in emissions from the road network, thus the advantage of using local information can be clearly seen.

In conclusion for the emission part, the approach of the FAIRMODE's tools, which offers a comparison between different inventories, using for example downscaled and local data, is useful. However, there is a large gap in resolution and methodology between local emission data and European downscaled emissions. It would be useful if FAIRMODE could provide guidelines on how to downscaling European emission data using national/regional information, e.g. using distribution of different types of local fireplaces, deployment of district heating networks, population density.

### 5.2 Concentrations

Temporally and spatially resolved concentration calculations were based on the emission inventory of the Air Quality Management Association of Eastern Sweden for the year 2015 (Segersson et al., 2017).

The annual mean concentrations of NO2 and PM10 were calculated using a Gaussian air quality dispersion model and a wind model, both part of the Airviro Air Quality Management System (Apertum IT AB, Sweden, https://www.airviro.com). The long-range transport (LRT) contributions were taken from measurements at rural sites outside the calculation domain. To reduce the time for computations while still maintaining a high resolution in the vicinity of roads and point sources, a quadtree (Finkel and Bentley, 1974) receptor grid was used. The receptor grids had a coarser resolution (around 500 m × 500 m) in rural areas without any emission sources, successively increasing in areas with emissions to a maximum of around 35 m × 35 m along major roads and close to stacks.

The Gaussian model estimates air pollution concentrations 2 m above ground level or 2 m above roof height in urban areas, and handles the influence of buildings on dispersion by using a roughness parameter. This results in underestimated concentrations in street canyons with heavy traffic. Therefore, a secondary set of concentrations maps were produced where, on top of the Gaussian modelled concentrations, was nested an open-street pollution model (OSPM), also part of the Airviro system.

The Gaussian model can reproduce annual NO2 and PM10 at background monitoring sites (see Figure E, supplementary information, top Figure E) but for traffic sites adjacent to motorways and in street canyons, calculations with a street model are needed in order to pass the Modelling Quality Objective (MQO) of the DELTA tool (see Figure E, centre and bottom).

**Fig. 6:**
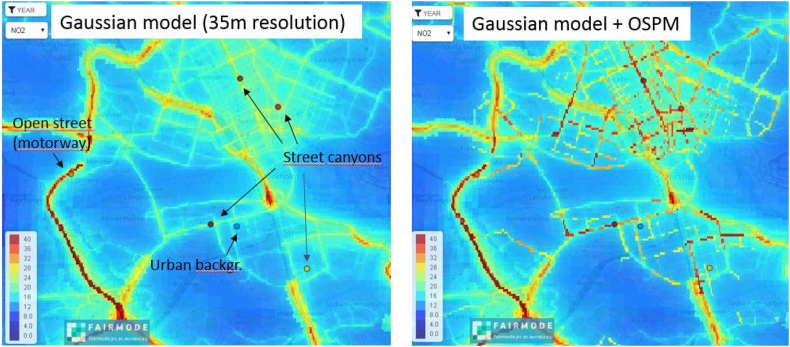
Annual NO2 concentration maps for Stockholm inner city, calculated with the Gaussian model and the nested OSPM model, respectively. Observed annual concentrations at monitoring stations are denoted within the black circles.

Fig. 6 shows annual NO2 concentration maps calculated with the Gaussian model (left) and the nested OSPM model (right), respectively as well as observed concentrations at local monitoring stations.

From this analysis, it is confirmed that one needs high resolution and high quality emission data, as well as dispersion models that are fit for purpose, for local simulations. At the same time the use of the DELTA tool facilitates the benchmarking of air quality models application.

## 6 Conclusions

In this paper we have presented the FAIRMODE pilot initiative. FAIRMODE is a European network of air quality modelling experts working on harmonization, improvement and guidance on the use of air quality models in the context of the Air Quality Directives. In the frame of FAIRMODE, the ‘pilot initiative’ has been launched, to assess the benefits of the methodologies developed in FAIRMODE to improve modelling activities. This initiative, on one side, provides regional and local authorities with tools and training for performing air quality assessment and management practices activities; and on the other side provides feedback to improve the FAIRMODE tools.

The paper has confirmed how the FAIRMODE tools can be used to support authorities in benchmarking and possibly improving their modelling capability.

In terms of emissions, we have shown that:

- FAIRMODE provides methodologies and tools (i.e. DELTA-emis-tool and Composite mapping tool for emissions) to compare downscaled (TOD) and bottom up (BUP) emission inventories;
- Differences in inventories (in terms of total emissions and spatial distribution) can be easily identified, with a first indication towards the main reason for these discrepancies (i.e. due to emission factors or activity level differences);
- A deeper and more detailed discussion of the discrepancies between TOD and BUP emission inventories (e.g. in terms of temporal distribution, which is not currently part of the FAIRMODE tools) can contribute to improve the understanding of emission processes and methods to better represent real emissions, as well as to provide permanent improvements to both current TOD and BUP inventories;
- For this to happen, a close interaction and collaboration between TOD and BUP developers needs to happen, and FAIRMODE gives the room for these types of discussions.

Identified limitations of the current FAIRMODE tools for emissions include:

- To analyse and understand discrepancies between TOD and BUP emission inventories, detailed knowledge of emission inventories (e.g. metadata of emission inventories, input data and methods) is needed and at the moment is not included in the FAIRMODE tools;
- The DELTA-emis-tool is useful to compare emission inventories and identify discrepancies. However, within the context of specific air quality planning, BUP and TOD inventories will rarely be compared. Rather, it is conceivable to use the tool to validate large scale TOD inventories using detailed local BUP ones;
- All the analyses of the DELTA-emis-tool are based on the assumption that emission factors and activity rates of the BUP and TOD inventories correspond well for at least one substance. This substance has to be selected as the “norming” substance for the diagrams. This assumption should be verified before using the tool and the “norming” substance should be selected accordingly. As the tool has a default “norming” substance and does not force the user to actively select one, it should always be documented that this assumption has been (successfully) checked and the respective substance been actively selected when applying the tool;
- For spatially differentiated data on the “European Composite Map Platform”, copyrights pose a problem. Maps on the platform are labelled only by a short name and there is no indication of ownership or copyrights. As a consequence, the right to provide emission maps on the platform is not always granted;
- The possibility of adding together/combine emissions from different source sectors would be useful, a functionality that is currently lacking on the composite platform.

In terms of modelling of concentrations, the pilot exercise has shown that:

- The Model Quality Objective and its implementation in the DELTA-conc-tool are mature approaches that can be used to evaluate the quality of modelled air quality data, compared to measured concentrations;
- The Composite Mapping tool offers the possibility to compare concentration maps produced by different modelling teams. The impact of different model resolutions can be evaluated, as well as the evaluation of differences, inconsistencies and discontinuities at national or regional borders, due to e.g. different input data and modelling methods. The tool can also be used to visually check differences between model results and measurements;
- Differences in concentration results from different modelling applications over the same domain can trigger discussion among modelling teams about e.g. model setups, applied boundary conditions, data assimilation schemes or underlying emission inventories;
- This kind of discussion will lead to an enhanced collaboration amongst the air quality scientific community leading to an overall improvement and harmonization of modelling capabilities and methods in Europe.

Identified limitations of the current FAIRMODE tools for the assessment of modelled the concentrations include:

- When applying the DELTA-conc-tool, it cannot be stressed enough that one is not assessing the quality of a model but rather of the entire modelling chain of a specific application (including meteorological data, emissions, the model, the modeller, and possibly data assimilation methodology);
- The data of several years could not be studied at once and graphs summarising the results for several years was not possible to be created by the DELTA-conc-tool. The benefits of allowing the study of multi-year data, needs to be considered on the further development of the tool;
- the “European Composite Map Platform” offers currently no possibility to query data base entries associated with the maps, in order to answer important questions in the operational use, such as whether or not model data has been assimilated, which input data have been used, etc …

In conclusion, we think that the ‘pilot’ initiative is bringing added value to the work of air quality modellers. It helped/is helping researchers to meet in a regular base, exchange ideas, discuss and compare the results of their studies via the DELTA-tools and the composite mapping tool. In this way, researchers and practitioners can realize possible discrepancies and implement corrections to improve the development of the local emissions and air quality modelling.

Nevertheless, apart from the benefit to the direct participants to the exercise, we think there are also benefits to practitioners and researchers “outside” this activity. In terms of emissions, we are aware about the fact that different emission communities are currently working partly in an “isolated” way (i.e. European, national and local emission inventories are not harmonized and consistent). We think that the harmonized approach proposed to compare emission inventories in the frame of the ‘pilot’ exercise can bring benefits by helping to “bridge the gap” and allowing to “speak the same language” to different communities. A practical example of this “linking” could be through the “composite mapping on emissions”, which could in the future lead to an emission inventory based on “collated” locally estimated information, to possibly improve emission inventories at EU scale. For concentrations, the MQI has been used in this exercise to benchmark modelling results at different scales, from national to regional to local. As modelling is not always validated (as shown in the FP7 APPRAISAL project results, Thunis et al., 2016a, Thunis et al., 2016b) and if validated, sometimes by using specific ad-hoc procedures, we still think it is important to perform and disseminate activities using the MQI, to further stress the importance of validating air quality modelling using a consistent, harmonized and comparable procedure across Europe.

The pilot initiative will now move to the second phase of the work (on planning), but it remains open to any regional/local authorities willing to follow the proposed steps for emission and concentration benchmarks.
