# Supplementary material for: Supporting the improvement of air quality management practices: The “FAIRMODE pilot” activity
Source: J Environ Manage. 2019 Sep 1;245:122–30. doi: 10.1016/j.jenvman.2019.04.118 (PMC6584326; doi:10.1016/j.jenvman.2019.04.118)
Supplement: Multimedia component 1 [file mmc1.docx]

# The delta tools

Two versions of the DELTA tool exist in FAIRMODE: for 1) emissions and 2) concentrations. In general terms, the emission tool allows for relative comparisons of two inventories to identify inconsistencies and further analysis, without providing absolute information on the quality of the emission inventories. The concentration tool (‘DELTA-conc-tool’) on the contrary provides an absolute benchmark against an “objective” standard, i.e., the observed concentration values. More details on the tools are presented below.

The ‘DELTA-emis-tool’ on emissions is an Interactive Data Language (IDL) based tool designed to screen and compare emission inventories. The tool provides diagrams and indicators to support the comparison of locally produced vs Europe-wide (or downscaled) emission estimates, for a given common geographical area (i.e., specific model domain, city, region or country). The tool contains four methods for comparing emission inventories; 1) bar-plot, for the comparison of pollutant emissions across sectors; 2) diamond-diagram, for the quantification of the differences between emission inventories in terms of activity data and emission factors; 3) per-capita diagram, for the comparison in term of emissions per capita; 4) pollutant ratio diagram, based on the ratio of specific pollutants. For more details about the theoretical background and use of these four methods, we refer to Guevara et al., (2016) and Thunis et al., (2016). In this manuscript, the assessment of management practices is built on the use of the bar-plot and diamond-diagram. The tool has currently five EU-wide emission inventories available for the comparison and benchmarking with local emission inventories. These inventories are EC4MACS (valid for 2007; Bessagnet et al., 2016), TNO_MACC-II (valid for 2009) and TNO_MACC-III (valid for 2011, Kuenen et al., 2014), JRC (valid for 2010, Trombetti et al., 2018) and EMEP (valid for 2015; EMEP-GNFR; CEIP, 2018). The tool was originally designed as a flagging system to identify inconsistencies in emission inventories, and to evaluate the reasons for these inconsistencies. The usefulness of the tool has been reported in different studies (e.g., Guevara et al., 2016; López-Aparicio et al., 2017; Trombetti et al., 2018; Madrazo et al., 2018), where the comparison of emission inventories compiled through different approaches has increased the understanding of emission estimation processes to ultimately improve both local and downscaled inventories.

The concentration benchmarking tool (here called ‘DELTA-conc-tool’) is an IDL based modelling evaluation software which is currently used for supporting modelling groups across Europe in the diagnostics and evaluation of their air quality modelling results with respect to measured data according to the AQD (2008) data quality objectives. The tool makes use of paired data of modelled and observed surface pollutant concentrations and computes different statistical indicators as well as the so-called *Modelling Quality Indicator* (MQI). A modelling result is considered as “good enough for policy application” when the MQI fulfils the *Modelling Quality Objective* (MQO): MQI ≤ 1. In addition the main statistical indicators (i.e., bias, RMSE, correlation coefficient), are summarized in appropriate statistic tables. The tool can also visualize them on a wide range of plots (scatter, time series, Taylor and target diagrams), providing an overview of the quality of modelling results with respect to observations. Currently the Modelling Quality Objective methodology implemented in DELTA-conc-tool (Thunis et al., 2012) is under discussion in the frame of the CEN (European Committee for Standardization) to be used as a standard for evaluating the quality of air quality modelling applications.

The DELTA-conc-tool has been already extensively used for modelling validation (see <http://fairmode.jrc.ec.europa.eu/wg1.html> for more details).

# The MQI

# The overall benchmarking evaluation procedure of MQI works on paired series of model and observed values of the respective pollutant for year-long periods. It investigates the model capabilities by introducing an overall indicator, namely, the modeling quality indicator (MQI), taking into account the measurement uncertainty of each pollutant. The measurement uncertainty parameters currently used in the FAIRMODE methodology have been previously presented, and are different for coarse particulate matter and nitrogen dioxide. MQI defines the deviation between measured (O) and modeled (M) values at a given i time (hour or day) as a factor of the measurement uncertainty and a scaling factor that indicates the stringency of the objective to be satisfied (e.g., a scaling factor of 2 means the allowed deviation should be within a factor of 2 of the measurement uncertainty gap):


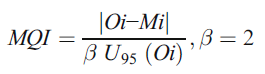


Oi and Mi are the observed and modeled values, respectively, U_95_ is the 95th percentile measurement uncertainty of the observed concentration level, and β is the coefficient that scales the proportionality of the bias to the measurement uncertainty. MQI can be generalized to a yearly time series as follows:


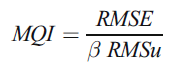


where RMSu is the root mean square of the measurement uncertainty and RMSE is the root mean square error between model and measured concentrations. For annual values, Oi and Mi are substituted in the formula by the observed and modeled annual mean, respectively, and the uncertainty is calculated at the 95th percentile upon the annual mean measured concentration. The modeling quality objective (MQO) is the criterion for the value of the MQI to be satisfied for satisfactory model performance in terms of air quality representation for reporting applications (minimum level of quality). MQO is fulfilled if the MQI is less than or equal to unity for at least 90% of the available monitoring data (Thunis et al., 2012b).

# Key challenges and priorities of the participants

In the case of Helsinki, traffic emissions lead to an exceedance of the annual limit value for NO2 with a risk of exceedance of the PM10 daily limit value in some busy street canyons; and the emissions from domestic wood burning increase PM2.5 and B(a)P concentrations in single-family residential areas.

In Stockholm, road traffic emissions give rise to exceedances of NO2 and PM10 in some busy street canyons as well as in the vicinity of some very busy motorways. As in the case of Helsinki, domestic wood burning is an important source of PM2.5 and B(a)P in single-family residential areas.

In the monitoring stations located in the urban part of Sofia Municipality major contribution to the average annual and daily concentrations of PM10 are the transport and domestic heating. For B(a)P, the contribution to concentrations is predominantly from the household sector during the heating period (75% on a calendar year basis).

In Dublin, Ireland, the following pollutants and sources are of concern:

- NO2 concentrations close to heavily traffic streets. This is a result of the relatively high proportion of diesel vehicles in the Irish vehicle fleet.
- Particulate matter (PM_10_, PM_2.5_) and BaP from residential solid fuel use for home heating

The principal aims of the Emilia Romagna pilot participation is to identify inconsistencies between Europe wide downscaled emission inventories and local/regional ones, as well as to evaluate the performance of regional air quality using the methodology implemented in DELTA-conc-tool, to check if the modelling application fulfils the model quality requirements set. Also, more in general, Italy has significant concentrations of NO2, PM10 and O3, leading to widespread exceedances of yearly (for NO2) and daily (for PM10 and O3) limit values, due to traffic, residential heating, and agriculture.

Croatia has examined pressure of emissions on its five main cities (Zagreb, Osijek, Slavonski Brod, Rijeka and Split) trying to explain differences resulting from diverse geographical position, climate and anthropogenic activities in addition to the influence of the extent of the city area and the population size.

The most problematic air pollutants in Slovenia are PM10 and O3. High PM10 levels are due to widespread use of wood for domestic heating in technically outdated stoves and boilers, together with low wind speed conditions in basins and valleys, accompanied by pronounced long-lasting temperature inversions. High O3 levels in Slovenia typically relate to widespread Mediterranean episodes, where the highest O3 levels in Slovenia are measured in Primorska region with higher temperatures, more sunshine hours and under the greatest influence of trans-boundary pollution.

The main sectors affecting the air quality in Athens are road transport, small combustion (domestic wood burning) and navigation. Regarding domestic heating, the economic crisis has led to the increase of biomass burning replacing conventional heating methods such as boilers, doubling in this way PM10 and CO emissions (from 2012 onwards) and leading to frequent particulate pollution episodes under winter temperature inversion conditions. As far as O3 episodes are concerned, road transport and port activities play the most important role as ozone precursors emitted in the region drive the photochemical cycle (Fameli and Assimakopoulos, 2016). It is important to note that Athens is located at the Eastern-Mediterranean region a most affected part of Europe in terms of photochemical and particulate pollution episodes as it is a crossroads of pollutants from the Sahara, Asia and Central-Northern Europe, biogenic sources as well as high temperatures and sunlight.

The objective of the modelling for Hessen state was to provide background concentrations and source apportionments in a high spatial resolution to be used in air quality planning for cities throughout the state.

# Additional Figures and Tables


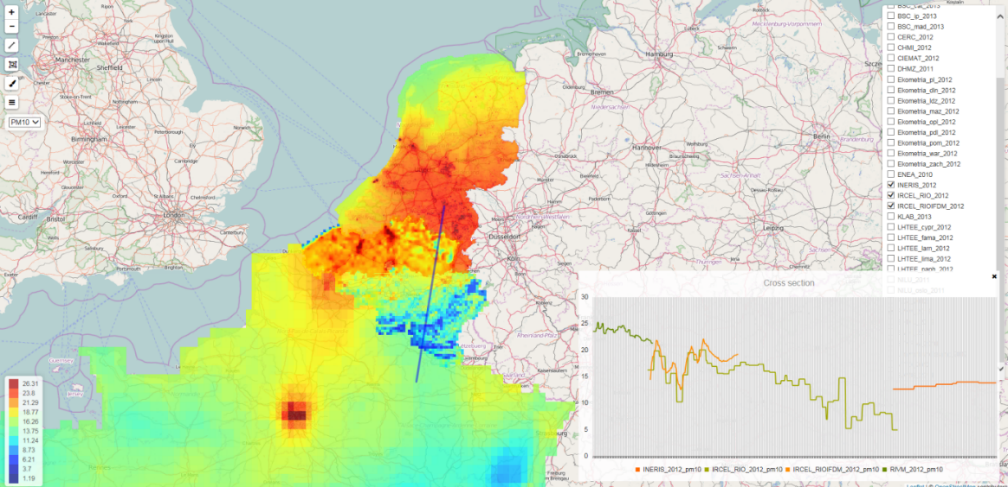


Figure A: Snapshot of the Composite Concentration Map. PM10 concentration maps are visible for the Netherlands, Belgium and France. Concentration profile (right bottom) along a user defined transect in the map (blue line). Inconsistencies at the Belgian-French border become visible.


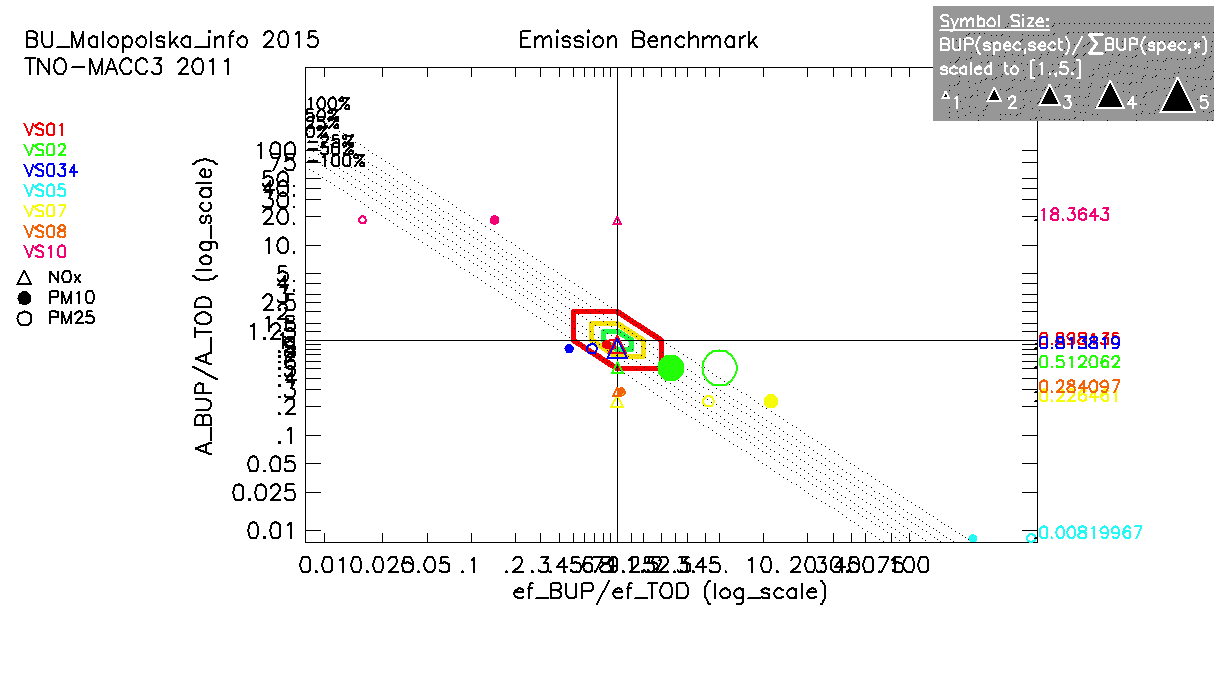


Figure B: 'diamond plot' for emissions in the Malopolska region.


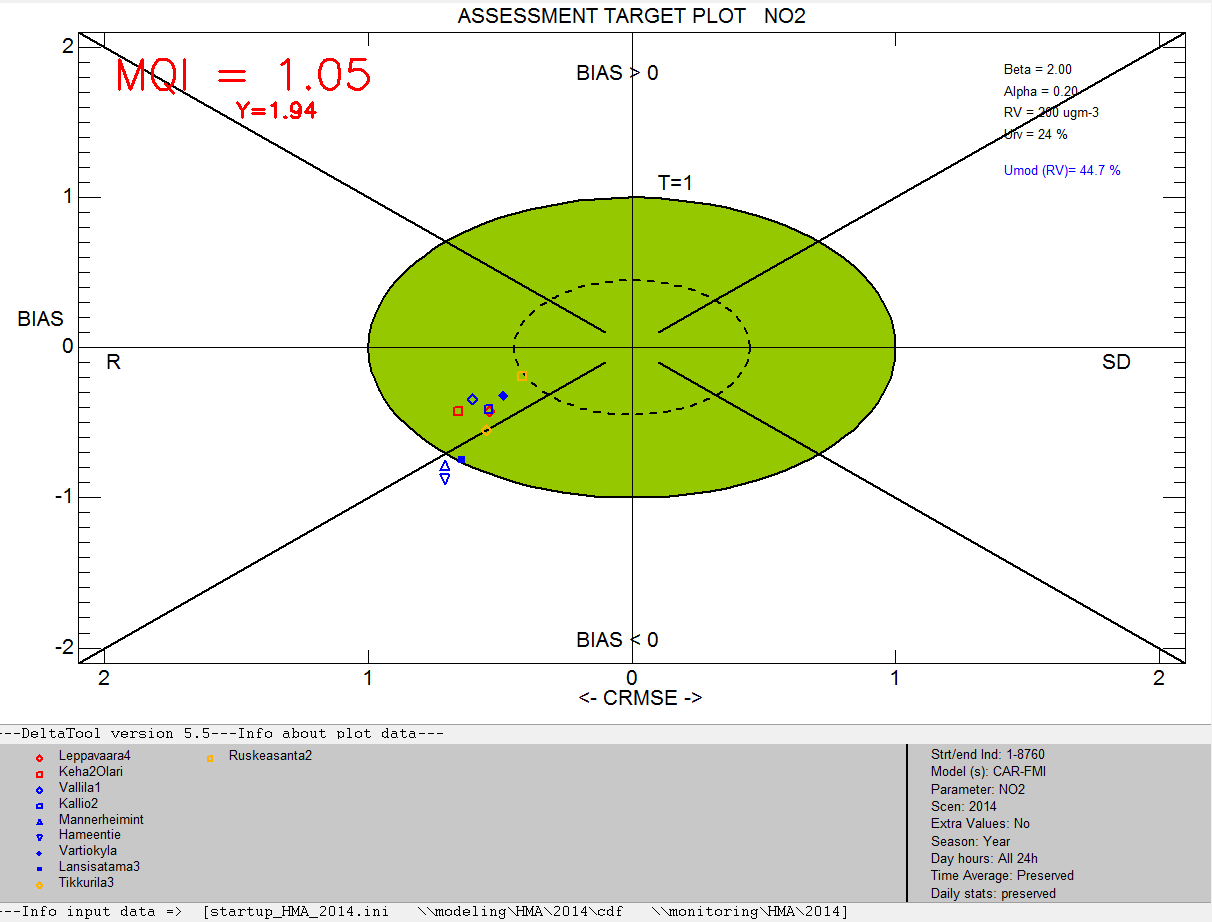


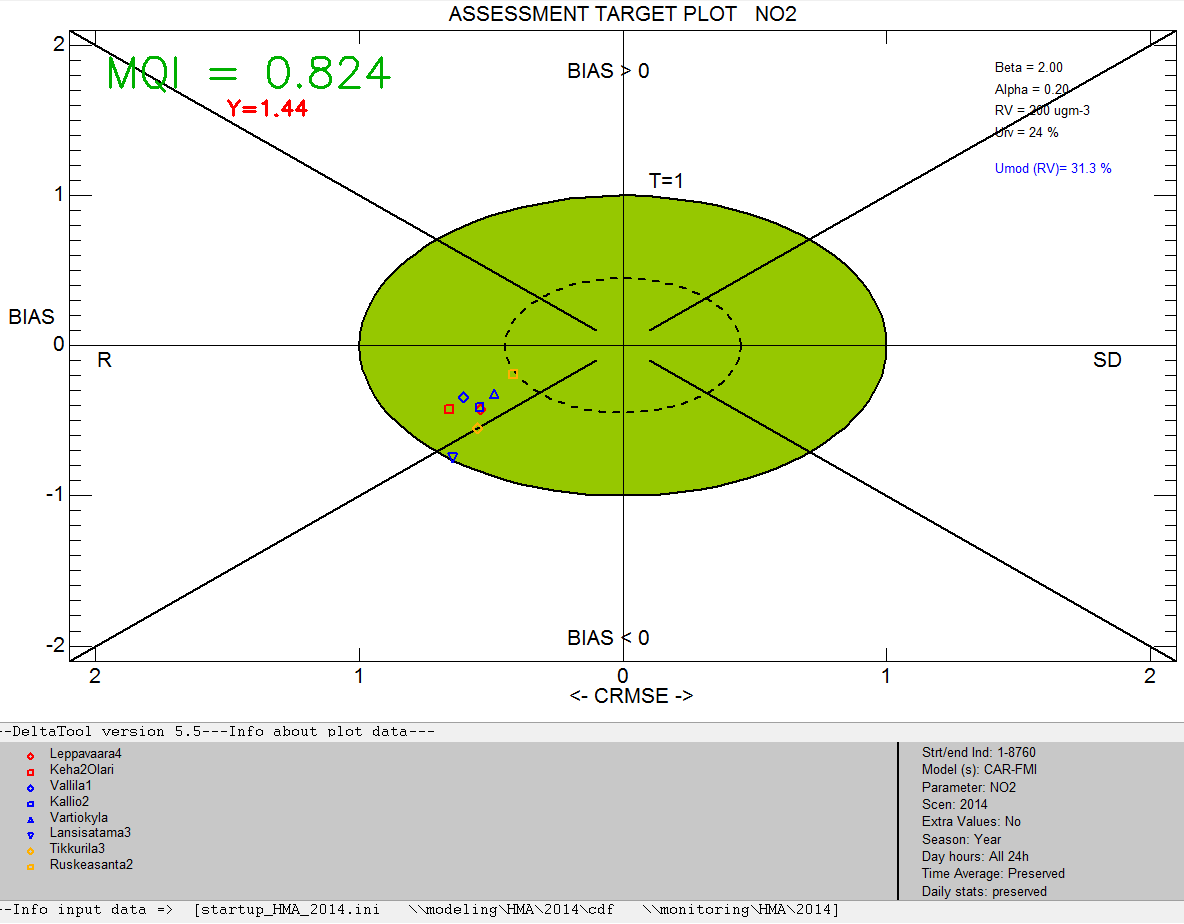


Figure C: Target plot of hourly NO2 concentrations in 2014 in Helsinki with all 10 stations (up) and with relevant 8 stations (down).








Figure D: Bar plots from the FAIRMODE DELTA tool for emissions. Top diagram: ratio between Stockholm local emissions and EMEP. Bottom diagram: ratio between Stockholm local emissions and TNO MACC 3. ENr = residential combustion, TRA = road traffic, TRAd = road traffic diesel exhaust, TRAp = road traffic petrol exhaust, TRAw = road traffic non-exhaust.


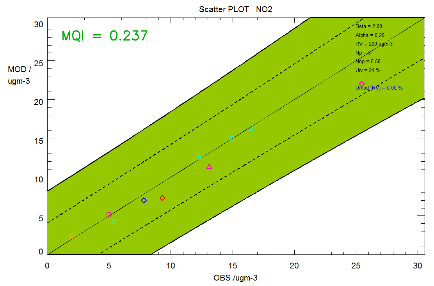


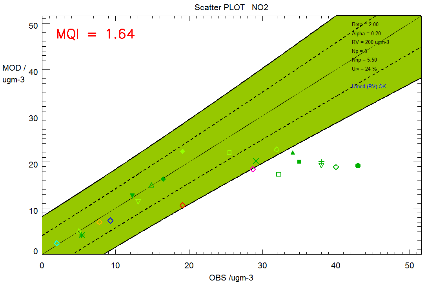


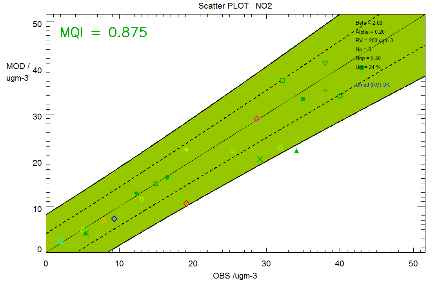


Figure E: Scatterplots and Modelling Quality Index (MQI) of modelled versus observed NO2 concentrations in Stockholm County, (top) Gaussian model and background monitoring sites; (centre) Gaussian model and background as well as traffic monitoring sites; (bottom) Gaussian+OSPM model and background as well as traffic monitoring sites. When the MQI is lower or equal to one, the MQO is attained.

Table A: some details of the participants to the pilot activity, with key features of their modelling application.

| **Country** | **Pilot city/region** | **Model name** | **Model type (Eulerian, …)** | **Model spatial resolution** |
| --- | --- | --- | --- | --- |
| Sweden | Stockholm city/region | Airviro | Gaussian + OSPM | 35-500m (variable grid) |
| Italy | Milan city | Calpuff | Lagrangian puff | 500m |
|  |  | Austal2000 | Lagrangian particle | 5-20m |
| Ireland | Dublin city | ADMS urban | Gaussian | 100m |
| Greece | Athens | WRF/CAMx | Eulerian | 6km (Greece)  2km (Athens) |
| Bulgaria | Sofia | AIRMOD | Gaussian | 500m |
| Finland | Helsinki | CAR-FMI | Gaussian | 20-500m |
| Italy | Emilia Romagna region | Ninfa (COSMO/CHIMERE) | Eulerian | 5km |
| Poland | Malopolska Region | GEM-AQ | Eulerian, on-line | 2.5km (now), previous 5km |
| Germany | Hessen state | RCG (REM/CALGRID) | Eulerian | 500m |
| Slovenia | Country | ALADIN-SI/CAMx | Eulerian | 4.4km |
| Croatia | Country | LOTOS-EUROS | Eulerian | 7km |
|  |  | ADMS-urban | Gaussian | 0.5km |
| Italy | ENEA | AMS-MINNI | Eulerian | 4km |

Table B: Definition of the SNAP sectors.

| **SNAP** | **Sector Name** |
| --- | --- |
| 1 | Combustion in energy and transformation industries |
| 2 | Non-industrial combustion plants |
| 3 | Combustion in manufacturing industry |
| 4 | Production processes |
| 5 | Extraction and distribution of fossil fuels and geothermal energy |
| 6 | Solvent use and other product use |
| 7 | Road transport |
| 8 | Other mobile sources and machinery |
| 9 | Waste treatment and disposal |
| 10 | Agriculture |

# References

Bessagnet, B., Pirovano, G., Mircea, M., Cuvelier, C., Aulinger, A., Calori, G., Ciarelli, G., Manders, A., Stern, R., Tsyro, S., García Vivanco, M., Thunis, P., Pay, M.-T., Colette, A., Couvidat, F., Meleux, F., Rouïl, L., Ung, A., Aksoyoglu, S., Baldasano, J. M., Bieser, J., Briganti, G., Cappelletti, A., D'Isidoro, M., Finardi, S., Kranenburg, R., Silibello, C., Carnevale, C., Aas, W., Dupont, J.-C., Fagerli, H., Gonzalez, L., Menut, L., Prévôt, A. S. H., Roberts, P., and White, L., 2016. Presentation of the EURODELTA III intercomparison exercise – evaluation of the chemistry transport models' performance on criteria pollutants and joint analysis with meteorology. Atmos. Chem. Phys. 16, 12667-12701.

Fameli, K.M. and Assimakopoulos, V.D., 2016. The new open Flexible Emission Inventory for Greece and the Greater Athens Area (FEI-GREGAA): Account of pollutant sources and their importance from 2006 to 2012. Atmos. Environ. 137, 17-37.

Guevara, M., López-Aparicio S., Cuvelier C., Tarrason L., Clappier A., Thunis P., 2016. A benchmarking tool to screen and compare bottom-up and top-down emission inventories. Air Qual. Atmos. Hlth. 10, 1-16.

Kuenen, J., Visschedijk, A.J.H., Jozwicka, M., Denier van der Gon, H.A.C., 2014. TNO-MACC_II emission inventory; a multi-year (2003-2009) consistent high-resolution European emission inventory for air quality modelling. Atmos. Chem. Phys. 14, 10963-10976.

López-Aparicio S., Guevara M., Thunis P., Cuvelierd K., Tarrasón L., 2017. Assessment of discrepancies between bottom-up and regional emission inventories in Norwegian urban areas. Atmos. Environ 154, 285-296.

Madrazo J., Clappier A., Belalcazar L., Cuesta O., Contreras H., Golay F., 2018. Screening differences between a local inventory and the Emissions Database for Global Atmospheric Research (EDGAR). J. Sci. Total Environ. 631-632, 934-941.

Thunis, P., Georgieva, E., Pederzoli, A., 2012. A tool to evaluate air quality model performances in regulatory applications. Environ. Modell. Softw. 38, 220-230.

Thunis P, Pederzoli A, Pernigotti D, 2012b. Performance criteria to evaluate air quality modeling applications. Atmosphetic Environment 59, 476–482.

Thunis, P., Degraeuwe, B., Pisoni, E., Ferrari, F., Clappier, A., 2016. On the design and assessment of regional air quality plans: The SHERPA approach. J. Environ. Manage. 183, 952-958.

Trombetti, M., Thunis, P., Bessagnet, B., Clappier, A., Couvidat, F., Guevara, M., Kuenen J., Lopez-Aparicio, S., 2018. Spatial inter-comparison of Top-down emission inventories in European urban areas. Atmos. Environ 173, 142-156.
